# The complete chloroplast genome of *Tugarinovia mongolica* Iljin (Asteraceae) from China

**DOI:** 10.1080/23802359.2021.1926356

**Published:** 2022-01-27

**Authors:** Lin Cong, Haiyan Jiang

**Affiliations:** Forestry College, Inner Mongolia Agricultural University, Huhhot, PR China

**Keywords:** *Tugarinovia mongolica*, chloroplast genome, next-generation sequencing

## Abstract

The complete chloroplast (cp) genome sequence of *Tugarinovia mongolica* Iljin has been characterized in this study. The cp genome is 153,475 bp in length, containing a large single-copy (LSC) region of 84,434 bp and a small single-copy (SSC) region of 18,715 bp, which were separated by a pair of 25,163 bp inverted repeat regions (IRs). There are 114 unique genes annotated, including 80 protein-coding genes, 30 tRNA genes, and four rRNA genes. The overall GC content is 37.62%. Further, phylogenetic analysis suggested that *Tugarinovia* is clustered to genus *Atractylodes*.

*Tugarinovia mongolica* Iljin, included in the China Species Red List, belongs to the monotypic genus (*Tugarinovia*) of Asteraceae family (Fu 1992). The species has a limited geographical range and declining populations in the western part of Inner Mongolia. Much attention have focused on the taxonomy, origin, and biology fields (Ma et al. 2000; Zhao 2000). However, there is no complete chloroplast (cp) genome of *T. mongolica* in GenBank database. Here, we first reported the complete cp genome of *T. mongolica*, and assessed its phylogenetic position within Carduoideae.

In this study, fresh leaves of *T. mongolica* were sampled from the urban area of Wuhai Ordos, Inner Mongolia province, China (40°03′56″N, 106°54′08″E). Voucher specimens were deposited at the herbarium of Inner Mongolia Agricultural University (voucher number: SLBHZW202008). The genomic DNA was extracted by the modified CTAB method (Doyle 1987) and then sequenced using Illumina-HiSeq 2000 platform (Illumina, San Diego, CA), with a 150 bp paired-end running. NovoPlasty was used to assemble the cp genome (Dierckxsens et al. 2017), with the cp genome of *Cynara cardunculus* var. *scolymus* as the reference (GenBank accession no. KM035764) (Curci et al. 2015). Then, the assembled sequence was annotated with GeSeq (Tillich et al. 2017). The annotated cp genome sequence has been submitted to NCBI with an accession number of MW429288.

The complete cp genome of *T. mongolica* is 153,475 base pairs (bp) in length. The canonical quadripartite structure consists of a large single-copy (LSC) region of 84,434 bp, a small single-copy (SSC) region of 18,715 bp, and two inverted repeat regions (IRs) of 25,163 bp. The new sequence possesses 114 unique genes, including 80 protein-coding genes, four rRNA genes, and 30 tRNA genes. Among them, four rRNA genes (i.e. *rrn16*, *rrn5*, *rrn4.5*, and *rrn23*), seven protein-coding genes (i.e. *rpl2*, *rpl23*, *ycf2*, *ndhB*, *rps7*, *rps12*, and *ycf1*), and seven tRNA genes (i.e. *trnI-CAU*, *trnL-CAA*, *trnV-GAC*, *trnI-GAU*, *trnA-UGC*, *trnR-ACG*, and *trnN-GUU*) are duplicated in the IR regions. The overall GC-content of the cp genome is 37.62%. The GC content of the LSC and SSC regions is 35.74% and 34.4%, respectively, whereas that of the IR regions is 43.1%.

To further determine its phylogenetic position, phylogenetic analyses using 17 Carduoideae cp genomes with two *Sonchus* species as outgroups were performed. After alignment by MAFFT v7 (Katoh and Standley 2013), we found model ‘GTR + I+G’ is the fittest model for phylogenetic construction by jModelTest (Posada 2008). Finally, a Bayesian inference (BI) phylogenomic tree was performed in MrBayes v3.2.3 (Ronquist and Huelsenbeck 2003). The Markov chain Monte Carlo (MCMC) algorithm was run for 1,000,000 generations with trees sampled every 500 generations. Results in this study indicated that *T. mongolica* is clustered to genus *Atractylodes* (Figure 1).

**Figure 1.**
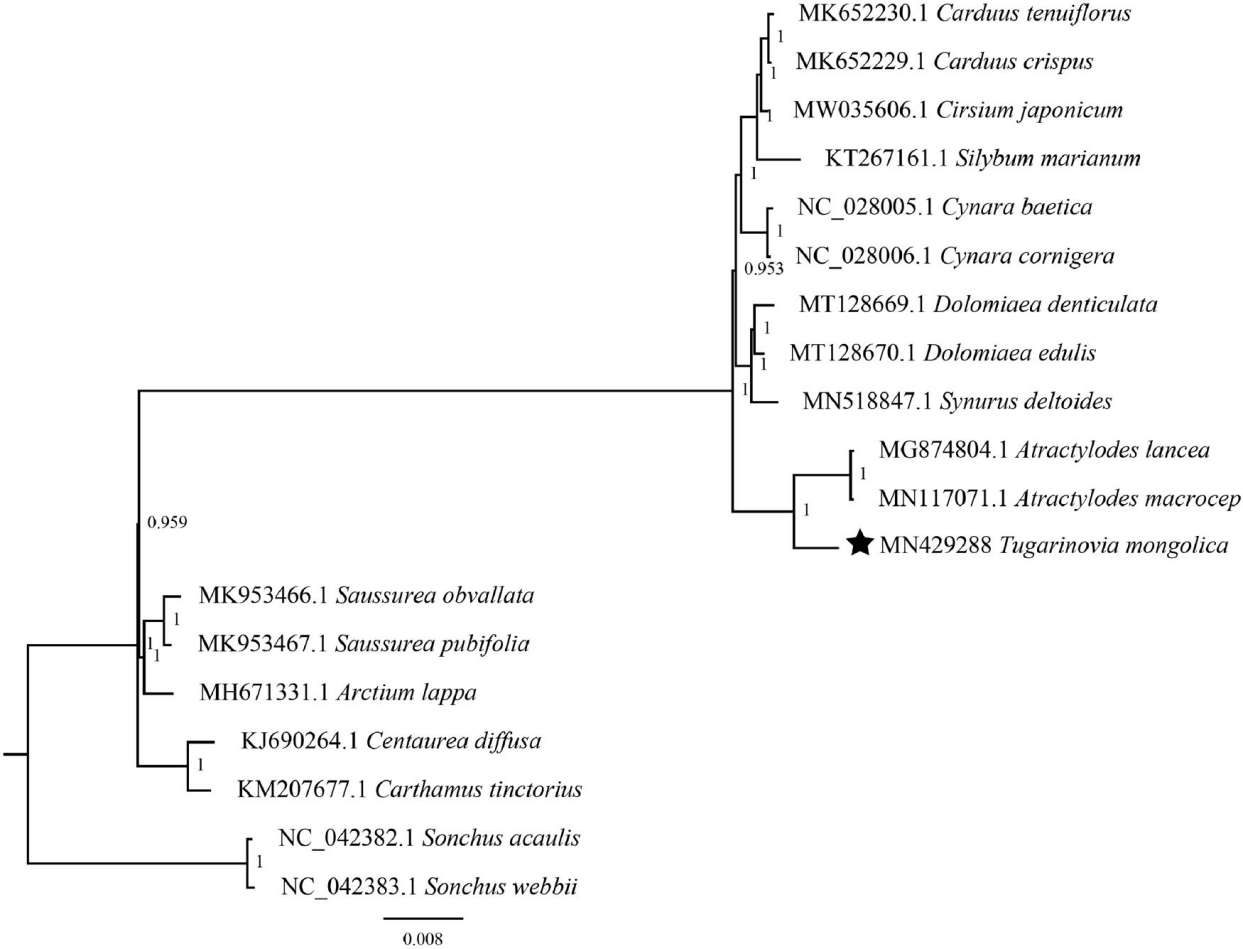
Phylogeny of 17 Carduoideae species based on chloroplast genome sequences. *Sonchus acaulis* and *S. webbii* were selected as outgroups. BI posterior probability is indicated for each branch.

## Data Availability

The genome sequence data that support the findings of this study are openly available in GenBank of NCBI at https://www.ncbi.nlm.nih.gov/ under the accession no. MW429288. The associated BioProject, SRA, and Bio-Sample numbers are PRJNA700100, SAMN17824457, and SRR13649040, respectively.
